# Childhood leukemias in Mexico: towards implementing CAR-T cell therapy programs

**DOI:** 10.3389/fonc.2023.1304805

**Published:** 2024-01-18

**Authors:** Juan Carlos Bustamante-Ogando, Alejandrina Hernández-López, César Galván-Díaz, Roberto Rivera-Luna, Hugo E. Fuentes-Bustos, Angélica Meneses-Acosta, Alberto Olaya-Vargas

**Affiliations:** ^1^Immunodeficiencies Research Laboratory and Clinical Immunology Department, Instituto Nacional de Pediatría, Mexico City, Mexico; ^2^Laboratorio 7 Biotecnología Farmacéutica, Facultad de Farmacia, Universidad Autónoma del Estado de Morelos, Universidad Autónoma del Estado de Morelos (UAEM), Cuernavaca, Morelos, Mexico; ^3^Consejo Nacional de Humanidades Ciencias y Tecnologías, CONAHCYT, Mexico City, Mexico; ^4^Oncology Department, Instituto Nacional de Pediatría, Mexico City, Mexico; ^5^Hematopoietic Stem Cell Transplantation and Cell Therapy Program, Instituto Nacional de Pediatría, Mexico City, Mexico

**Keywords:** childhood leukemia, Mexico, immunotherapy, cellular therapy, CAR-T cells, global access, gene therapy, cell therapy

## Abstract

Leukemias are the most common type of pediatric cancer around the world. Prognosis has improved during the last decades, and many patients are cured with conventional treatment as chemotherapy; however, many patients still present with a refractory disease requiring additional treatments, including hematopoietic stem cell transplantation. Immunotherapy with monoclonal antibodies or cellular therapy is a promising strategy for treating refractory or relapsed hematological malignancies. Particularly, CAR-T cells have shown clinical efficacy in clinical trials, and different products are now commercially approved by regulatory agencies in the USA and Europe. Many challenges still need to be solved to improve and optimize the potential of these therapies worldwide. Global access to cell therapy is a significant concern, and different strategies are being explored in the middle- and low-income countries. In Mexico, leukemias represent around 50% of total cancer diagnosed in pediatric patients, and the rate of relapsed or refractory disease is higher than reported in other countries, a multi-factorial problem. Although significant progress has been made during the last decades in leukemia diagnosis and treatment, making new therapies available to Mexican patients is a priority, and cell and gene therapies are on the horizon. Efforts are ongoing to make CAR-T cell therapy accessible for patients in Mexico. This article summarizes a general landscape of childhood leukemias in Mexico, and we give a perspective about the current strategies, advances, and challenges ahead to make gene and cell therapies for leukemia clinically available.

## Introduction

Leukemia is the most common cancer in children, its diagnosis and treatment have favorably changed in the last 50 years. Many factors contribute to a better prognosis, including improvements in diagnosis, biologic classification, and the development of new therapeutics, causing overall survival rates to improve from 30% in the 1960s to over 80% in this decade in developed countries. Low-risk pediatric leukemias currently have a survival of 95% in the ideal scenario, although refractory and relapsed leukemia still cause high mortality and disease-burden (1). Notable is the emerging field of cellular immunotherapy, where Chimeric Antigen Receptor (CAR) T cells show encouraging results in hematologic malignancies (2).

Access to new diagnostic and therapeutic options is not equally available worldwide. In low- and middle-income countries (LMIC) like Mexico many challenges need to be addressed in different areas, including clinical care, basic and clinical research, education, funding, and regulation to allow access to more cell and gene therapies.

In this paper, we describe some important aspects of the past and current healthcare status for pediatric leukemias in Mexico, review the basic and clinical aspects of CAR-T cell therapy, and discuss the challenges to make this therapy available.

## Leukemia treatment in Mexico

Leukemias account for around 50% of newly diagnosed cancer in children (3, 4). Acute lymphoblastic leukemia (ALL) is the most common type of childhood leukemia (83%), followed by acute myeloid leukemias (13.1%), chronic myelocytic leukemia (2.8%), and myelodysplastic syndromes (1%) (5). Between 1998-2018, the proportion of leukemias as a cause of pediatric deaths increased from 0.3% to 1.3% (6). It represents a public health problem with socio-economic and psychological impact on patients and families. In general, the mortality rate is higher when compared with developed countries (3).

Mexico has a population of 130 million, the healthcare services are diverse. Health insurance is limited, and costly procedures like cancer treatment and HSCT are challenging to fund. The IMSS (Instituto Mexicano del Seguro Social) provides healthcare to the formally employed citizens, the ISSSTE (Instituto de Seguridad y Servicio Sociales de los Trabajadores del Estado) to the bureaucrats, and the SSA (Secretaría de Salud) covers population without a formal employment. Less than 7% of Mexicans have Private healthcare (7). Currently, there are certified pediatric oncologists and certified centers in every State of the Country. However, disparities in access to diagnostic and therapeutic procedures exist among centers and public and private hospitals (3). Nine centers are accredited to perform HSCT in pediatric patients distributed in Mexico City, Puebla, Guadalajara, and Monterrey (8).

In Mexico, the poor prognosis of children with ALL is multifactorial including biological and sociocultural factors. Pivotal studies in Mexico identified nutritional problems as a significant adverse prognostic factor for patients with leukemia (9). It has been reported that these two factors were not significant for early mortality, but they condition a higher risk of infections during treatment, leading to increased overall mortality (10). Currently, nutritional interventions must be part of the clinical care for all children with leukemia.

Before 2005, pediatric cancer was not part of Mexico’s National Health Program. The introduction of a National Health Program in 2005 provided specific financial coverage for leukemia treatment and national treatment protocols based on risk stratification, leading to improved prognosis. It also allowed broader access to hematopoietic stem cell transplantation (HSCT) for relapsed and refractory leukemias, increasing survival from 16% to 50% (11, 12). A retrospective analysis for pediatric ALL treated between 2005 and 2015 showed an estimated overall 5-year survival rate of 61.8%; the annual number of treated children doubled from 535 in 2005 to 1,070 in 2015 (13). Several factors contribute to the low survival rates for pediatric ALL in Mexico, including specific molecular epidemiology, late diagnosis, low precision diagnostic methods, limited access to treatment, therapy-related toxicity, exposure to environmental risk factors, high frequency of early relapse, lack of collaborative work, insufficient involvement in clinical trials, and lack of continuous medical education (14–16).

Significant effort has taken place to improve the diagnosis and treatment of pediatric leukemias. In 2016, a collaborative program with St. Jude Hospital (USA) started, seeking to develop multi-site protocols for clinical care. An initial retrospective evaluation showed that 82% of patients were classified as high-risk leukemia at diagnosis and that tests for minimal residual disease or molecular classification were unavailable on 28% of patients, highlighting challenges for evaluation and classification at diagnosis (17). More than 57 centers are now enrolled in this initiative, and a National Program for Pediatric Leukemia Treatment (MAS-ALL18 AMG) exists for the first-line treatment protocols (18). Other initiatives, such as ONCOCREAN, are taking place to improve regional clinical care and access to more precise diagnosis and classification methods. HSCT programs are available in many parts of the country, and now is considered a standard of care in refractory/relapsed ALL. Although access to new therapies and clinical trials improves slowly, there are positive developments. In a recent study that included 41 Mexican patients with CD19+ ALL treated with blinatumomab, 78% showed a complete response (Unpublished). Additionally, 63% of them received haploidentical HSCT after blinatumomab, with a significant improvement in survival compared to conventional chemotherapy (19).

The National Council for Humanity, Science and Technology (CONAHCYT) launched an initiative in 2020 to fund basic and clinical research to improve the prognosis of pediatric patients with leukemia (PRONACES SALUD) and reduce the gap between different regions across the country. We highlight three research areas from this initiative: 1) The national harmonization of flow cytometry tests for diagnosis and follow-up, 2) Improving knowledge about clinical and molecular epidemiology of Mexican patients with leukemia, 3) The development of a clinical program for CAR-T cell therapy (20).

Pediatric leukemia outcomes are sub-optimal in Mexico, especially for relapsed and refractory leukemias. The field is evolving rapidly worldwide and immune effector cell therapy, particularly CAR-T cells, is an area of great interest and opportunity for the upcoming years.

## Understanding the designs and clinical concepts of CAR-T cells

**Figure 1:**
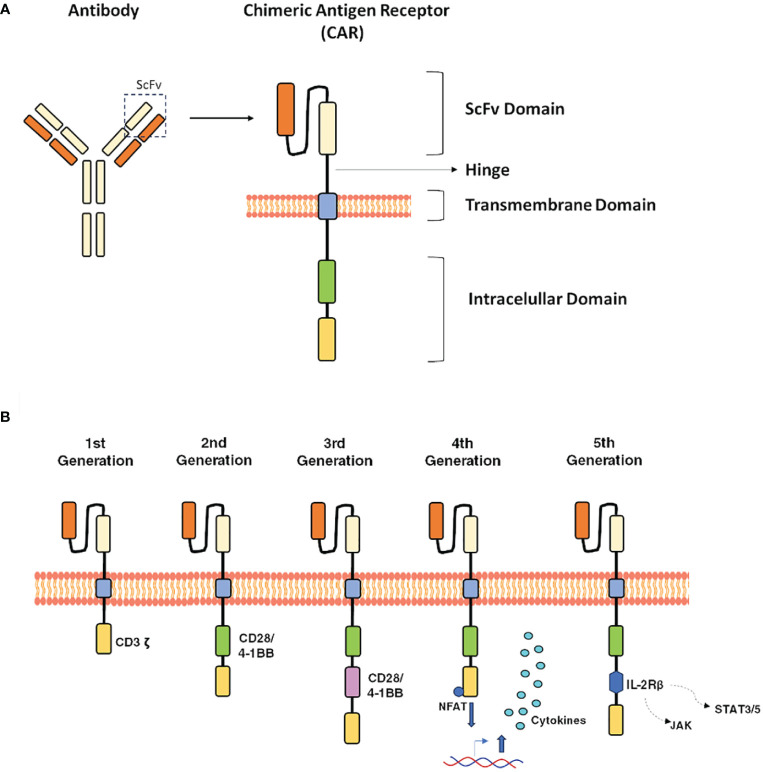
Main structure and evolution of CARs design. **(A)**, The core structure of a CAR highlighting the major components, 1) extracellular domain: antigen-recognition domain, a single-chain fragment variant (scFV), 2) space domain, 3) transmembrane domain, and 4) an Intracellular domain. **(B)**, the CAR evolution is classified into five generations: the first one, only contain the CD3ζ, the second one includes additional costimulatory signaling domains (CD28 or 4–1BB), while, the third-generation combine multiple co-stimulatory domains, such as CD28–41BB or CD28-OX40 and the fifth generation contain an extra intracellular domain. This CAR comprise truncated intracellular domains of cytokine receptors (IL-2Rβ) with a motif for binding transcription factors such as STAT-3/5.

CAR-T cells are genetically engineered T cells expressing a CAR directed against a specific target. In oncology, CAR-T cells are directed against tumoral antigens, enabling the destruction of cancer cells without needing an interaction between T and antigen-presenting cells (21). The CAR design is an essential part of the success of this therapy. CARs combine a monoclonal antibody’s specificity with T cells’ effector functions. CAR-T cells express engineered receptors composed of four main domains: 1) an antigen-binding extracellular domain, which is typically a single-chain variable fragment (scFv) of monoclonal antibody (mAb), 2) a spacer domain that provides flexibility and helps to position the CAR toward the target, 3) a transmembrane domain that contributes to the stability and promotes cytokine release, and 4) an intracellular domain which provides T cell activation and expansion signals (22, 23) (**Figure 1A**). In addition, to enhance the activity, persistence, and safety of CAR-T cell therapy, different intracellular scaffolds have been designed and currently classified into five generations (**Figure 1B**) (24–27).

The CAR-T cells have emerged as an effective therapy for treating relapsed and refractory (R/R) B-ALL in children and adults (27). CD19/CAR-T cell infusion treatment induced high complete remission (CR) rates in patients with poor prognosis and few therapeutic options in clinical trials and real-life treatment reports (27, 28). However, this therapy still has significant limitations, such as long manufacturing times, high costs, its autologous nature, and resistance mechanisms that could reduce its effectiveness (25). Despite encouraging responses, more than half of patients will experience a relapse in the long term (29). Consolidation with allogeneic HSCT could improve long-term outcomes (30).

Some approaches to enhance efficacy and avoid resistance to CAR-T cells involve alternative targets, such as CD20 and CD22, and the design of bi- and tri-specific CAR-T cells (31). Additionally, new platforms are developing to shorten the CAR-T cell manufacturing process (32). Another approach to increase the availability of CAR-T therapy involves knocking out the native human leukocyte antigen (HLA) and T-cell receptor (TCR) genes in T cells obtained from healthy donors to avoid compatibility issues, thus enabling the development of universal, off-the-shelf CAR-T cell therapies, which could expand accessibility to a more significant number of patients (25).

### Improving access to CAR-T cell therapy

By April 2022, there were 2,756 active cell therapy agents in immune-oncology research worldwide, a 36% increase compared to 2021. Remarkably, these therapies have yet to be geographically distributed significantly in recent years, with most clinical trials in the USA and a few European countries (33). Barriers to the wider clinical use of CAR-T cells include costs, patient health status, a limited number of treatment and manufacturing centers, and geographic distance (34).

India recently initiated a phase III clinical trial at a Mumbai Hospital (35, 36) with a strategy to manufacture CAR-T cells on-site, aiming to reduce ten-fold costs compared to commercial products. Brazil has authorized more than 18 clinical trials with Advanced Therapy Products since 2018, and in July 2022, the first CAR-T cell therapy clinical trial was authorized (37, 38). Initiatives for global access to CAR-T cell therapies are ongoing in different parts of the world (39). A Latin American Consortium initiated in collaboration with Caring Cross in 2022, where specialists from Brazil, Argentina, Chile, Mexico, and Colombia regularly meet and discuss advances and challenges for national and regional implementation (40).

### Regulatory framework

CAR-T cell research and clinical use must join a regulatory framework. 2017, the first CAR-T cell therapy received FDA approval (41). Currently, six CAR-T cell products are approved by the FDA (USA)/EMA (Europe) for the treatment of hematologic malignancies (**Table 1**). Heterogeneity in regulation worldwide adds significant challenges to advancing gene and cell therapy. The FDA considers gene therapy products to be biologics, and the regulatory framework is issued by the Center of Biologics Evaluation and Research (CBER). EMA considers CAR-T cells as cellular products and regulates them under the Advanced Therapy Medicinal Products (ATMP) framework. Both agencies published regulatory guidelines that agree on the importance of controlling the quality of each element involved in CAR-T cell production (42, 43).

**Table 1:** Approved CAR-T cell products.

Generic Name	Tisagenlecleucel	Axicabtagene ciloleucel	Brexucabtagene autoleucel	Idecabtagene vicleucel	Lisocabtagene maraleucel	Ciltacabtagene autoleucel
**Commercial Name**	Kymriah	Yescarta	Tecartus	ABECMA	Breyanzi	Carvykti
**Company**	Novartis	Kite/Gilead	Kite/Gilead	Bristol Myers Squibb/Bluebird bio	Juno/Bristol Myers Squibb	Janssen Biotech, Inc.
**Indication**	Treatment of pediatric and young adult patients (age 3- 25 years) with B-cell ALL that is refractory or in second or later relapse. Adult patients with (r/r) LBCL after two or more lines of systemic therapy including DLBCL, HGBL and DLBCL arising from FL.	Treatment of adult patients with (r/r) LBCL after two or more lines of systemic therapy, PMBCL, HGBL, and DLBCL arising from FL.	Treatment of adult patients with (r/r MCL).	Treatment of adult patients with R/R MM after four or more prior lines of therapy.	Treatment of adult patients with r/r DLBCL after two or more lines of systemic therapy, HGBL, PMBCL, and FL grade 3B.	Treatment of adult patients with relapsed or refractory multiple mieloma.
**Target**	CD19	CD19	CD19	BCMA	CD19	BCMA
**Approved FDA/EMA**	August 17^th^, 2017/August 18^th^ 2018.	October 18^th^, 2017/August 23^rd^, 2018.	July 24^th^, 2020/December 14^th^, 2020.	March 26^th^, 2021/August 18^th^, 2021.	February 5^th^ 2021/April 4^th,^2022.	February 28^th^, 2022/May 25^th^, 2022.
**Cost per dose**	$475,000	$373,000	$373,000	$545,000	$470,940	$504,344
**Orphan drug**	——	——	Yes	Yes	——	Yes
**Profit 2022**	$139 M	$337 M	$82 M	$125 M	$55 M	$55 M

Currently, there are only six FDA/EMA approved products for different cancer types. ——, No.

World Health Organization (WHO) also published a document recognizing that this type of product requires specific regulation and classifies CAR-T cells as ATMP´s (44).

Regulatory frameworks from the FDA, EMA, and WHO may serve as a basis for establishing regulations worldwide. Regulation must be dynamic to facilitate accessibility while maintaining high-quality standards. Some of the difficulties in Mexico and Latin America in establishing a clear regulatory framework include a) the lack of specific laws or regulatory documents regarding gene and cell therapies, b) the absence of expert committees to evaluate these products and, c) the lack of financial support for the research and development of regulation.

## Overcoming limitations to enhance CAR-T cell therapy accessibility in Mexico

Interest in CAR-T cell therapy has increased in Mexico in the last few years. Currently, there is only one clinical trial, led by the Autonomous University of Nuevo León, approved by the Mexican Regulatory Agency COFEPRIS for treating adults with CD19-positive leukemia or lymphoma. Notably, in early 2023, their group successfully imported a clinical-grade lentiviral vector for the first time in Mexico (45). Our group is developing a clinical trial for treating pediatric leukemia with CD19 CAR-T cells using an on-site semi-automated manufacturing process in a closed system. This manufacturing process has shown previously effective and reproducible cellular and clinical results (46).

### Costs

The cost of CAR-T cell therapy is currently estimated between US$373,000 and $475,000 per infusion, not including patient care expenses (47). There is a need for cost-effective strategies and efforts to optimize patient access (48).

In resource-limited settings, where many other healthcare problems need to be solved, the cost of CAR-T cell therapies is a significant barrier. Commercial CAR-T cells with centralized manufacturing have very high costs (49). Point-of-care manufacturing reduces costs significantly while maintaining good quality standards. The use of automated CAR-T cell manufacturing equipment has been demonstrated to be feasible in HIC and LMIC (35, 46). A recent study from India demonstrates the feasibility of a decentralized automated process of anti-CD19 CAR-T cells with fulfillment of all standard criteria assays for clinical application at a significantly reduced manufacturing cost of US$35,107 (excluding the cost of the lentiviral vector, which may vary from $5,000 to $28,889 per patient) (35). Real-world data on manufacturing costs in Mexico is lacking, but our estimates are around $30,000 per product. Costs of clinical care, apheresis, management of adverse reactions, and monitoring will add to the final treatment costs.

This high financial burden will need to be analyzed within the healthcare systems and the real impact these therapies will have on the general population (49). It is necessary to use different economic approaches to evaluate these therapies and see the potential long-term benefits (50, 51). For every child who does not survive cancer, there is a loss of 70 productive years for the Society (6). We performed a cost-benefit analysis using the social return on investment method, and we estimate that if 21 pediatric patients with refractory/relapsed leukemia are successfully treated with CAR-T cells, there is a social return of $5.43 for each $1 invested in the funding (unpublished). CAR-T cells may represent a cost-effective option for pediatric R/R B-ALL if a one-year survival above 70% is achieved (52).

### Regulatory framework

In Mexico, a robust regulatory framework is needed. There are issues that require our attention: a) general regulation for the clinical use of genetically modified cells and tissues, b) the regulation for the import/export, use, and manufacture of viral and non-viral vectors for clinical use, c) regulation of the manufacturing process, d) guidelines and regulation of specialized centers for clinical use of cell and gene therapies. In 2023, the Mexican Health Regulatory Agency (COFEPRIS) established for the first time a group of experts in advanced therapies from different fields, including academic, medical, scientific, pharmaceutical, and regulatory areas. The aim is to develop an initial regulatory framework.

### Access to clinical-grade vectors

Another limiting factor for CAR-T cell therapy is the accessibility clinical-grade viral vectors due to their high cost. In the future, local or regional manufacturing of GMP clinical-grade viral vectors may help to improve accessibility and reduce costs. Our group, in collaboration with Triovance, designed and produced a research-grade humanized lentiviral vector encoding for anti-CD19 CAR. Ongoing studies at the Faculty of Pharmacy at the Universidad Autónoma del Estado de Morelos are evaluating the CAR-T cell production processes using this vector (transduction, cell culture, cell expansion, and modified-T cells functionality) in a pre-clinical cellular model.

### Developing clinical care CAR-T cell programs

While cellular product manufacturing is a significant component of CAR-T cell therapy, specialized clinical centers and multi-disciplinary personnel are also needed to treat patients (53, 54). Starting to develop local and national clinical guidelines for CAR-T cell therapy will be beneficial to this field, as well as looking for accreditations from International standards (i.e. FACT) which strengthen our ability to manufacture cells and treat patients according to international quality standards, broadening also international collaborations (55).

### HSCT programs as a pathway to successful clinical CAR-T programs implementation

HSCT is formally available in Mexico since 1995, and currently, around 15 centers have experienced and well-established programs in public and private hospitals, with variable capacity to perform autologous and allogeneic procedures from compatible related and unrelated, cord blood, or haploidentical donors (8, 56). There are several reports of the Mexican HSCT experience, which can be comparable with the success in other developing and developed countries (57). Mexican reports have shown how specific transplantation models adapted to our reality can maintain good clinical results at reduced costs (58). This infrastructure and experience may serve as a basis for developing local and National immune effector cell programs to broaden the impact and reach more patients. There is a need of establishing thoughtful, focused, and efficient care models to achieve maximum benefits in resource-limited areas.

## Discussion

The historical journey of pediatric leukemia diagnosis and treatment in Mexico shows remarkable improvements during the last decades and the capacity for change within healthcare systems.

CAR-T cell therapies have demonstrated efficacy and safety in recent clinical trials, leading to exponential progress in research in this field. In the upcoming years, these therapies will likely become part of conventional treatments for refractory or relapsed leukemias.

Currently, CAR-T cell therapy is not an essential or cost-effective treatment for emerging economies. There are also opinions on the need for more rationale for providing this therapy at the public expense of patients (50). However, with the early clinical successes and current advancements, much funding has been pouring into the field, and rapid improvements in equipment, reagents, and methods will eventually start to push down the costs. Research, non-profitable models, and academic collaborations between public and private institutions may help generate real-world data in the coming years.

Several key issues must be addressed to improve global access in Mexico, including infrastructure and material resources, training of human resources, reducing supplies and manufacturing costs, gaining clinical experience through clinical trials, and establishing an appropriate regulatory framework. Support from health authorities, collaborative efforts of academic, scientific, and private institutions, and increased investment in health and research are all indispensable. Specific research on the Mexican population is also required to determine whether specific characteristics might impact the outcomes of these treatments.

Implementing cellular therapies in Mexico and Latin America is feasible and necessary. With the potential growth of these therapies in the upcoming years, we need to start working and tackling challenges. There is infrastructure and previous experience in cancer diagnosis and treatment, with high-quality transplant programs and blood banks at the national level, which will serve as a basis for implementing CAR-T cell therapy programs.

It is necessary to increase awareness and knowledge about cell and gene therapy and to encourage investment in research and healthcare. Initial steps are ongoing, and the goal is clear: making these therapies a reality for children around the globe. Different groups are now looking for options to implement CAR-T cell and other immune effector therapies in Mexico, and we believe this is a time of opportunity to establish national and international collaborations and share ideas to achieve this goal and benefit more patients in the future.

## Data availability statement

The original contributions presented in the study are included in the article/supplementary material. Further inquiries can be directed to the corresponding author.

## Author contributions

JB-O: Conceptualization, Writing – original draft, Writing – review & editing. AH-L: Conceptualization, Writing – review & editing. CG-D: Writing – review & editing. RR-L: Writing – review & editing. HF-B: Writing – review & editing. AM-A: Writing – review & editing, Conceptualization. AO-V: Conceptualization, Writing – original draft, Writing – review & editing.
